# Genome-wide studies in prostate cancer poised liquid biopsy as a molecular discovery tool

**DOI:** 10.3389/fonc.2023.1185013

**Published:** 2023-08-24

**Authors:** Nicholas Lo, Housheng Hansen He, Sujun Chen

**Affiliations:** ^1^ Princess Margaret Cancer Centre, University Health Network, Toronto, ON, Canada; ^2^ Department of Medical Biophysics, University of Toronto, Toronto, ON, Canada; ^3^ West China School of Public Health, West China Fourth Hospital, and State Key Laboratory of Biotherapy, Sichuan University, Chengdu, China

**Keywords:** prostate cancer, liquid biopsy, deep genomic sequencing, genome-wide methylation analysis, disease progression

## Abstract

Liquid biopsy is emerging as an intriguing tool in clinical disease detection and monitoring. Compared to a standard tissue biopsy, performing a liquid biopsy incurs minimal invasiveness, captures comprehensive disease representation, and can be more sensitive at an early stage. Recent genome-wide liquid biopsy studies in prostate cancer analyzing plasma samples have provided insights into the genome and epigenome dynamics during disease progression. In-depth genomic sequencing can offer a comprehensive understanding of cancer evolution, enabling more accurate clinical decision-making. Furthermore, exploring beyond the DNA sequence itself provides opportunities to investigate the regulatory mechanisms underlying various disease phenotypes. Here, we summarize these advances and offer prospects for their future application.

## Main

Treatment options for metastatic lesions of prostate cancer (PCa) are limited, and resistance to androgen signalling inhibitors (ASI) is ultimately inevitable (1–3). Detecting aggressive disease while it is still manageable and understanding the underlying biology are clinical imperatives. The standard invasive tissue biopsy procedure for PCa diagnosis poses a risk to the patient (4), is limited in the early stages of disease (5), and is impractical for longitudinal disease monitoring. Liquid biopsies utilizing body fluids such as blood, urine and saliva and analyzing biomaterials in circulation show promise for revolutionizing tumour profiling and monitoring practices. It contributes to understanding the signals determining threshold tumour development (6), highlights metastatic markers, and provides complementary information for treatment response (7, 8). In addition to the biomarker potential, it begins to serve as a method for evaluating mechanisms behind therapy resistance (9).

Characterization of metastatic PCa remains scarce, and previous studies are limited in scale and depth (9–11). Four recent studies (7, 12–14) analyzed the genome-wide genetic and epigenetic landscape using blood samples and brought deep biological insights associated with disease progression. In this mini-review, we summarize the key findings from these genome-wide studies and their implications for the potential applications of liquid biopsy. A concise overview of liquid biopsy research in prostate cancer was included to offer a more comprehensive context for our discussion.

## Liquid biopsy analytes commonly used in the clinical practice

**Figure 1:**
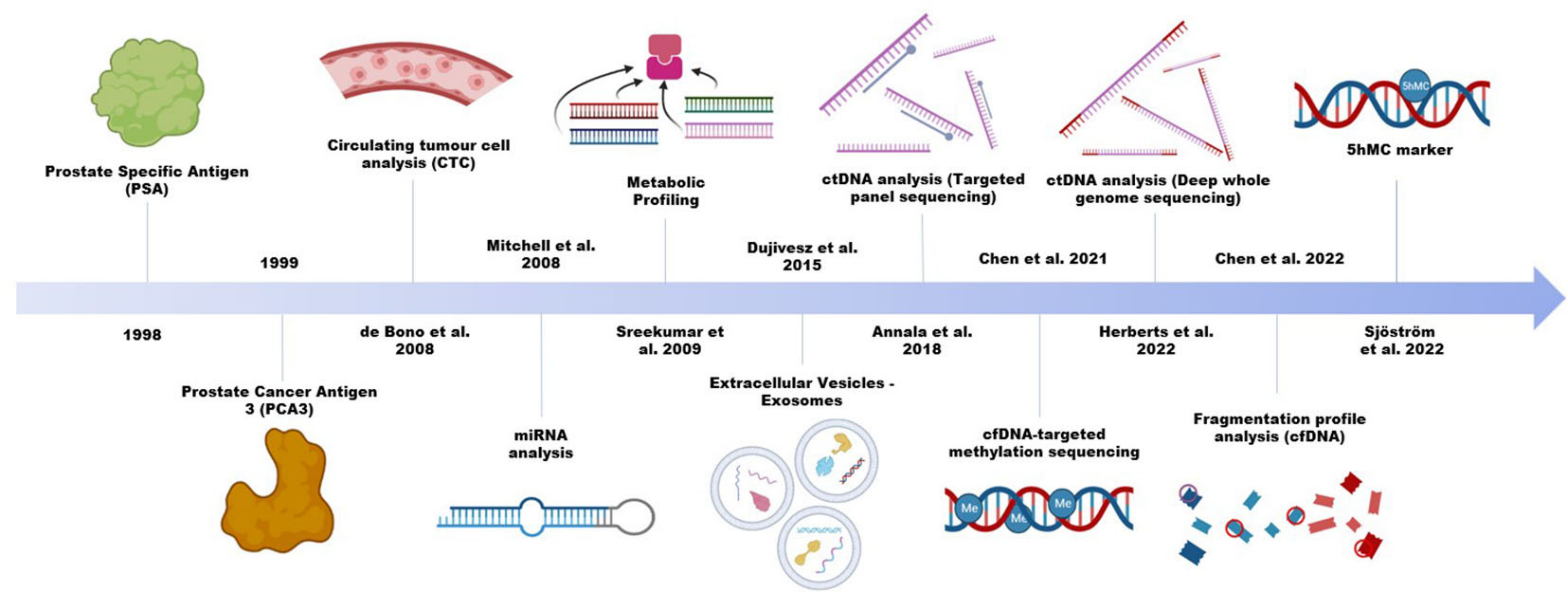
Application of various liquid biopsy technologies in prostate cancer.

Circulating tumour cells (CTC), extracellular vesicles, and membrane free biomolecules, including various types of nucleic acids and proteins, constitute the most commonly used analytes for liquid biopsy. In prostate cancer, a single protein biomarker, the prostate cancer specific antigen (PSA) remains active in clinical practice, despite its tendency to overdiagnose (**Figure 1**) or overemphasize the severity of low grade and slow growing tumours (15). PSA is a highly sensitive marker, but it is limited for specific detection in patients. There is in fact limited evidence for the practicality of PSA in a primary care setting (16). Thus, many efforts were instead devoted to improving diagnostic accuracy, with the most studied being the urine-based test of long non-coding RNA (lncRNA) prostate cancer antigen 3 (PCA3). The test was approved by the US Food and Drug Administration, and unlike PSA, it shows moderate sensitivity and adequate specificity in differential diagnosis of PCa and non-PCa (17). However, it remains controversial in terms of the degree of additional clinical benefit it can provide (18–21). For RNAs to be robustly analyzed in liquid biopsies, they need to survive the RNase-rich extracellular environment. Certain RNA species, such as the microRNAs (miRNA), exhibit greater stability, can be abundant with high specificity in patient plasma and are increasingly explored (22, 23). The formation of RNA-protein, RNA-DNA and RNA-lipid complexes are potential mechanisms mediating this increased stability of endogenous RNA transcripts. Alternatively, encapsulation in extracellular vesicles (EV) can help stabilize the transcripts, as in the case of PCA3. In 2016, an exosomal RNA assay became commercially available to help detect aggressive disease while reducing unnecessary biopsies (24, 25). Meanwhile, research on other types of cargo in EVs, such as DNA, is on the rise (26).

CTC is another important liquid biopsy biomarker in clinical settings and the CellSearch system for CTC enumeration was FDA approved in 2010 (27). While multiple studies including clinical trials have validated the usefulness of CTC for prognostication and disease monitoring, the majority of current studies have focused on its applications in late-stage disease (28–30). An apparent limitation of their use in clinical and laboratory settings is their low detection rate at early stages of the disease (31). With the CellSearch system, the percentage of localized prostate cancer patients with detectable CTC in a 7.5ml blood sample ranges from 5% to 27%, and the median count can be as low as 1 (32–35). As potential clinically relevant predictors of future metastasis, many studies have taken efforts to improve overall detection. Using microfluidic devices, Stott et al. and (36) were able to achieve detection in approximately half of their localized patient cohorts, with medians of 95 and 4.5 cells per mL of peripheral blood, respectively (37). Additionally, combination of the CellSearch system with apheresis technology dramatically increases the volume of blood analyzed (mean 59.9 ml) and improves the recovery of CTCs (mean 12,546) in metastatic PCa, showing great promise in analyzing localized diseases.

## Liquid biopsy in disease detection and monitoring in recent prostate cancer research

With minimal invasiveness, liquid biopsies are most well studied as biomarkers. Being able to capture a more holistic view of the disease is another attractive advantage of liquid biopsy. It is particularly important for managing metastases, as they cannot be represented by individual lesions and are difficult to biopsy. In contrast to CTC, ctDNA can be obtained more readily from patient plasma without the need for rare cell type enrichment procedures and can be more sensitive. It allows the detection of prognostic and predictive genomic alterations in driver genes, including AR, TP53, and those in the DNA repair pathways (9, 38–40). Specifically, blood-based identification of DNA damage repair (DDR) defects can help uncover potential candidates for DDR-directed therapies and immunotherapy, which might be overlooked when relying on primary tissue samples (41, 42). Herberts et al. (12) further showed that single-matched tissue biopsy failed to identify the dominant clone detected in plasma, potentially misinforming clinical decision-making.

With limited detectable mutations and low concentration at early stage, research focus was redirected towards advanced metastatic castration-resistant prostate cancer (mCRPC) to aid in prognostication and provide guidance for targeted therapies (30, 43–47). A study by Stover and colleagues (48) applied a novel NGS panel for evaluating patient-derived models, allowing for somatic variant detection over time across several prostate cancer-associated genes. This was found to be useful not only for primary tumours, but also for CTCs and cfDNA (48). In clinical practice, classification of patients as plasma tumour DNA positive or negative was done using an orthogonal approach designed to utilize known information on heterozygous SNPs (47). Prior to treatment with abiraterone acetate, higher levels of gene alterations were found in mCRPC patients with a higher initial disease burden. Plasma changes over time established that a sample post-abiraterone acetate + glucocorticoid treatment could identify resistant clones more effectively than a pre-treatment liquid biopsy sample (47). On the other hand, epigenetic alterations, specifically DNA methylation, are thought to occur early in the progression of the disease and have a greater number of recurrent sites with detectable frequencies (49). These features make them attractive candidates for early cancer detection and have been extensively investigated (50, 51).

While liquid biopsy studies have traditionally made use of molecules like nucleic acids and proteins (52), emerging types of analytes, including lipids, glycans, and microbiomes, are being explored as potential biomarkers for prostate cancer (52–55). Studies on the blood microbiome have revealed distinctive signatures between major cancer types, indicating potential as a complementary diagnostic tool to ctDNA/ctRNA assays (56). The approach to screening is also evolving from single analytes to multi-gene panels, and now whole genome investigations are becoming more common. Genome-wide studies have advanced not only in size but also in depth, accuracy, and methodology. They now delve deeper into the underlying biology of diseases rather than solely focusing on biomarker discovery (7, 12–14).

For example, by capturing alterations not commonly present at the DNA level and beyond the tumours themselves, DNA methylation can offer additional layers of information (13, 14). This is particularly useful for early cancer diagnosis, for which the sensitivity is limited by the low amount of ctDNA and the even lower number of variable biomarkers available. Chen and colleagues (13) showed that fragmentation profiles inferred from the methylation sequencing data differ significantly between healthy control and localized samples, while Sjostrom et al. captured 5hmC alterations not detected in the DNA. The ability of DNA methylation to capture lineage-specific features can be further explored to facilitate the development of multi-cancer early detection tests (57). A study by Bjerre et al. found hypermethylation rates in ctDNA to be as high as 61.5% in *de novo* metastatic PCa patients. A shorter progression duration towards resistant PCa was also correlated with detection of ctDNA methylation (58). Practically, the detection process also appears to be minimally invasive, and has been found to be associated with higher rates of medical compliance and cost efficiencies (59). In terms of its supplemental monitoring capabilities, it can make up for what PSA assessments currently lack. ctDNA monitoring is currently in transition towards potential clinical implementation. ctDNA percent levels do not necessarily reflect the same tumour characteristics as current evaluation methods, which as previously mentioned, can provide more information alongside current popular markers (60). For AR-directed therapy regimens, the changes in monitored ctDNA levels may act as indicators for early cancer progression, thus warranting therapy alterations (61, 62). Overall, ctDNA methylation analysis is showing to be capable of being a valuable tool for both detection and cancer management.

## Liquid biopsy as a tool for molecular discovery

Additional models and approaches have also been utilized to overcome challenges such as low ctDNA content and cancer diversity. The use of patient-derived xenograft (PDX) mouse plasma helped define nucleosome pattern analysis frameworks that can distinguish mCRPC phenotypes with up to 97% accuracy (7, 63). Two high-performance models were developed to approximate the proportion of neuroendocrine prostate cancer (NEPC) and androgen receptor-positive prostate cancer (ARPC), as well as predict their presence. An analysis framework implementing a GC correction procedure for cfDNA fragmentation patterns was also developed to achieve sensitive cancer subtype prediction (63).

Sarkar and colleagues employed PDX models with corresponding tissue samples to establish computational frameworks that can infer transcriptional activity by analyzing the nucleosome positioning pattern of ctDNA (7). They were able to link variations in nucleosome organization to changes in histone modifications, chromatin accessibility, and transcription factor activity that are specific to diverse tumour phenotypes (7). Using plasma ctDNA, the transcriptional activities of key phenotype regulators, including hepatocyte nuclear factor 4 gamma (HNF4G), AR, and achaete-scute homolog 1 (ASCL1), were detected, and the results showed high consistency compared to those obtained from tissue multi-omic profiling. Furthermore, direct estimation of phenotype proportion revealed that diverse molecular subtypes often coexist.

As well, the utilization of liquid biopsy has moved beyond its biomarker discovery ability. With deep whole-genome sequencing on the plasma samples from mCRPC patients, Herberts et al. (12) showed that different dominant clones exist for individual metastatic lesions. These differences could only be captured by liquid biopsies rather than tissue biopsies. They identified clinically relevant alterations that are difficult for bulk tissue sequencing to resolve, such as subclonal whole genome duplications, prevalent and diversified AR alterations, and convergence on AR augmentation after potent ASI treatment.

Chen et al. (13) and Sjöström et al. (14) used liquid biopsy to evaluate the DNA methylation landscapes. Through the use of immunoprecipitation in tandem with sequencing, Chen et al. were able to distinguish diverse forms of methylation and provide genome-wide cell-free profiles for 5mC, the most common form of DNA methylation (13). The cell-free methylomes revealed alterations apart from the tumour itself, coupled with global hypermethylation and hypomethylation at pericentromeric regions for mCRPCs compared to localized diseases. Using these data, the authors further inferred copy number alteration and fragmentation profiles, which showed notable distinctions among various disease stages.

For 5mC to reverse, it must first be oxidized to 5hmC, a mark for activated and poised transcription. Counting only a fraction of the total DNA methylation and unable to be distinguished from 5mC by the widely used bisulfite conversion-based methods, 5hmC was poorly understood in PCa until recently (14, 64). Sjostrom et al. used biotin labelling to specifically enrich 5hmC and provide a global landscape with paired liquid and tissue biopsies (14). The 5hmC dynamics throughout PCa progression identify cancer hallmarks and provide an additional layer of prediction by capturing non-canonical alterations. PCa-specific 5hmC patterns can track lineage plasticity and can be used to predict tumour burden in circulation (14).

## Summary

The biomarker potential of liquid biopsies has been extensively explored. Although ctDNA has demonstrated success in disease monitoring and DNA methylation has shown promise in early cancer detection, there is currently no single method that is comprehensive enough to achieve sufficient clinical accuracy and stability in both scenarios. Application of ctDNA analysis is greatly restricted due to the limited number of tumor-specific mutations, especially for early cancer detection where the amount of shedded ctDNA is low. While measuring epigenetic alterations can provide more detectable features, it is impeded by technology and analytical limitations. Traditional chemical methods are more accurate, yet they are not as cost-effective and can result in loss of the already limited DNA materials available. Conversely, enrichment-based methods are susceptible to influence of sequence specificity and antibody effectiveness, leading to potential inaccuracies. Similarly, while RNA transcripts are more readily detectable, they are highly variable and present challenges in reproducibility. Certain types of RNA, such as miRNA and circRNA, have proven to be relatively stable and are gaining increasing attention in research.

Recent studies reiterated the necessity of using liquid biopsy to avoid potentially ill-informed clinical decisions and opened up new avenues towards developing more accurate multi-modality assays. Information beyond the ctDNA sequence itself, such as epigenetic alterations and fragmentation profiles, are reflective of gene regulatory patterns and can provide more detectable features, thus enhancing the potential for early cancer detection. Therefore, conducting multi-omics sequencing can improve sensitivities, while implementing a stringent analysis pipeline that uses multi-factor verification can reduce false positives and promote overall accuracy. Moreover, as evidence suggests the presence of unique features in liquid biopsy samples emerging, genome-wide strategies are now more commonly employed to facilitate unbiased biomarker discovery. Such comprehensive and in-depth analysis of liquid biopsies has also led to significant biological insights, establishing it as a powerful tool for molecular discovery. The continued research with liquid biopsy will no doubt yield stunning insights into disease biology and facilitate the development of more effective therapeutics.

## Author contributions

HH and SC conceived and designed the research. NL, HH and SC wrote, revised and approved the manuscript.
